# Clinical Efficacy of an Electronic Portal Imaging Device versus a Physical Phantom Tool for Patient-Specific Quality Assurance

**DOI:** 10.3390/life12111923

**Published:** 2022-11-18

**Authors:** Seung-Hyeop Baek, Sang-Hyoun Choi, Moo-Jae Han, Gyu-Seok Cho, Wonil Jang, Jin-Sung Kim, Kum-Bae Kim

**Affiliations:** 1 Department of Integrative Medicine, Yonsei University College of Medicine, Seoul 03722, Republic of Korea; 2 Research Team of Radiological Physics & Engineering, Korea Institute of Radiological & Medical Sciences, Seoul 01812, Republic of Korea; 3 Medical Physics and Biomedical Engineering Lab (MPBEL), Yonsei University College of Medicine, Seoul 03722, Republic of Korea; 4 Department of Radiation Oncology, Korea Institute of Radiological & Medical Sciences, Seoul 01812, Republic of Korea; 5 Department of Radiation Oncology, Yonsei Cancer Center, Heavy Ion Therapy Research Institute, Yonsei University College of Medicine, Seoul 03722, Republic of Korea; 6 Oncosoft Inc., Seoul 03787, Republic of Korea

**Keywords:** quality assurance, radiotherapy, EPID, portal dosimetry software

## Abstract

Pre-treatment patient-specific quality assurance (QA) is critical to prevent radiation accidents. The electronic portal imaging device (EPID) is a dose measurement tool with good resolution and a low volume-averaging effect. EPIbeam—an EPID-based portal dosimetry software—has been newly installed in three institutions in Korea. This study evaluated the efficacy of the EPID-based patient-specific QA tool versus the PTW729 detector (a previously used QA tool) based on gamma criteria and planning target volume (PTV). A significant difference was confirmed through the R statistical analysis software. The average gamma passing rates of PTW729 and EPIbeam were 98.73% and 99.60% on 3 mm/3% (local), 96.66% and 97.91% on 2 mm/2% (local), and 88.41% and 74.87% on 1 mm/1% (local), respectively. The *p*-values between them were 0.015 (3 mm/3%, local), 0.084 (2 mm/2%, local), and less than 0.01 (1 mm/1%, local). Further, the average gamma passing rates of PTW 729 and EPIbeam according to PTV size were 99.55% and 99.91% (PTV < 150 cm^3^) and 97.91% and 99.28% (PTV > 150 cm^3^), respectively. The *p*-values between them were 0.087 (PTV < 150 cm^3^) and 0.036 (PTV > 150 cm^3^). These results confirm that EPIbeam can be an effective patient-specific QA tool.

## 1. Introduction

Radiotherapy is one of the techniques to treat cancer, along with surgery and chemotherapy.

Currently, patient-specific radiotherapies, such as intensity-modulated radiation therapy (IMRT), volumetric modulated arc therapy (VMAT), stereotactic body radiation therapy (SBRT), and stereotactic radiosurgery (SRS), are widely used because these techniques have the advantage of a small margin and high treatment performance [1,2,3,4,5]. Performing patient-specific quality assurance (QA) and machine QA are important to improve treatment performance and prevent radiation accidents when treated with such a high dose and precision radiotherapy techniques.

Various tools are used for patient-specific QA to verify the treatment planning. Matrixx (IBA dosimetry, Schwarzenbruck, Germany) based on ionization chamber and MapCheck (Sun Nuclear, Melbourne, FL, USA) based on diodes are typical 2D array detectors used as patient-specific QA tools [6,7,8]. The 3D array detectors, such as ArcCHECK (Sun Nuclear, Melbourne, FL, USA), based on the diode, are also used for patient-specific QA [9]. These detectors have the following advantages: stability for a short time, dose linearity, and simple dose calibration. However, they have an average volume effect, poor spatial resolution, and require phantom setup time [6,10,11].

An electronic portal imaging device (EPID) attached to the linear accelerator (LINAC) has a good resolution and a low volume-averaging effect; moreover, its setup is extremely simple [11,12,13]. Hence, the EPID can overcome the limitations of the array detector [14,15]. Furthermore, several research groups have verified its suitability as a patient-specific QA tool compared to commercial array detectors [13,16,17]. Another research group evaluated the dosimetric characteristics of EPID [18,19,20,21]. The relationship between dose and pixel value was studied by Grein et al. [18]. Furthermore, McDermott et al. studied the dose-response and ghosting effects of EPID and reported that the response deviation was within 1% under clinical conditions [19]. Louwe et al. [20] studied the long-term response stability (23 months) and reported that the deviation was within 0.5%. Further, Greer et al. [21] noted that EPID is a suitable QA tool for IMRT and VMAT based on the results of nine characteristics, including dose linearity, field-size dependency, and response to dose rate.

Several research groups have studied about EPID to use the patient-specific QA tool, and in line with this trend Varian—a radiotherapy machine corporation—provides EPID-based patient-specific QA software to users [22]. However, Elekta, which is another radiotherapy machine corporation, does not provide QA software. Hence, each institution has developed in-house software to use EPID [11,23].

Recently, Elekta began providing EPID-based patient-specific QA software developed by DosiSoft. In this study, we evaluated the clinical efficacy of the newly installed EPID-based portal dosimetry tool along with the evaluation of dosimetric characteristics.

## 2. Materials and Methods

### 2.1. Dosimetric Characteristics of the a-Si EPID

This study used the amorphous silicon (a-Si) flat panel imager (iViewGTTM, Elekta, Stockholm, Sweden) attached to the Elekta InfinityHD linear accelerator (Elekta, Stockholm, Sweden). It has a resolution of 1024 × 1024 pixels and a detection area of 41 × 41 cm^2^ at a fixed source-to-detector distance (SDD) of 160 cm. The EPID image acquired at SDD 160 cm is automatically converted to SDD 100 cm.

Four items were measured using 6 MV to evaluate the dosimetric characteristics of a-Si EPID. The measurement conditions for each item are summarized in Table 1. The acquired EPID image was analyzed using MATLAB R2021a (MathWorks, Natick, MA, USA) and DoseLab Pro software (version 6.8.0, Mobius Medical, Houston, USA).

#### 2.1.1. Linearity

We evaluated the linearity of the EPID response with an increasing monitor unit (MU) based on the following iso-center *pixel value*:

(1) $$Pixel\;value_{\left(isocenter\right)}=\frac{Raw\;pixel\;signal_{\left(isocenter\right)}}{Pixel\;sensitivity\;factor}$$

#### 2.1.2. Relative Output Factor (ROF) Depending on Field Size

We calculated ROF based on the field size (*FS*). The Semiflex ionization chamber (S/N 1278, PTW31010, Freiburg, Germany) was placed in a 2D water phantom filled with water. The *ROF* of *EPID* and ionization chamber (*IC*), depending on the field size, were calculated using Equations (2) and (3), respectively, and normalized based on 10 × 10 cm^2^.

(2) $$ROF_{FS}^{EPID}=\frac{PV_{FS\left(f\right)}}{PV_{FS\left(ref\right)}}$$

(3) $$ROF_{FS}^{IC}=\frac{M_{FS\left(f\right)}}{M_{FS\left(ref\right)}}$$

where f corresponds to each measured field size and ref denotes 10 × 10 cm^2^.

#### 2.1.3. Dose Rate Dependency

We measured 100–600 MU/min at 100 MU/min intervals to evaluate the difference in *EPID* responses depending on the dose rate (*DR*). The difference based on the dose rate normalized to 400 MU/min was calculated using Equation (4).

(4) $$Difference_{DR}^{EPID}=\frac{\left(PV_{DR\left(400\right)}-PV_{DR\left(dD/dt\right)}\right)}{PV_{DR\left(400\right)}}\times 100\left(\%\right)$$

where dD/dt denotes dose rate.

#### 2.1.4. Beam Profile: Flatness and Symmetry

We measured the in-line and cross-line profiles. First, two Semiflex 3D chamber (S/N 143069 and 143070, PTW31021, Freiburg, Germany) were used as a field chamber and reference chambers, respectively. The profile was measured using these ionization chambers placed in a PTW BeamScan water phantom (PTW, Freiburg, Germany). The flatness and symmetry of the measured profile were calculated using PTW MEPHYSTO mc^2^ software (PTW, Freiburg, Germany).

The EPID images acquired at SDD 160 cm were analyzed using DoseLab Pro software. The flatness and symmetry were calculated using Equations (5) and (6), respectively, based on the International Electrotechnical Commission (IEC) 60976 protocol [24].

(5) $$\mathrm{Flatness}\left(\%\right)=\frac{D_{max}}{D_{min}}\times 100$$

where $D_{\max}$ and $D_{\min}$ denotes the maximum and minimum doses with the beam profile at a depth of interest.

(6) $$\mathrm{Symmetry}\left(\%\right)={\left[\frac{D_{\left(x\right)}}{D_{\left(-x\right)}}\right]}_{max}\times 100$$

where $D_{\left(x\right)}$ denotes the dose at a point off the central axis by *x* distance.

### 2.2. Portal Dosimetry Software Commissioning

**Figure 1:**
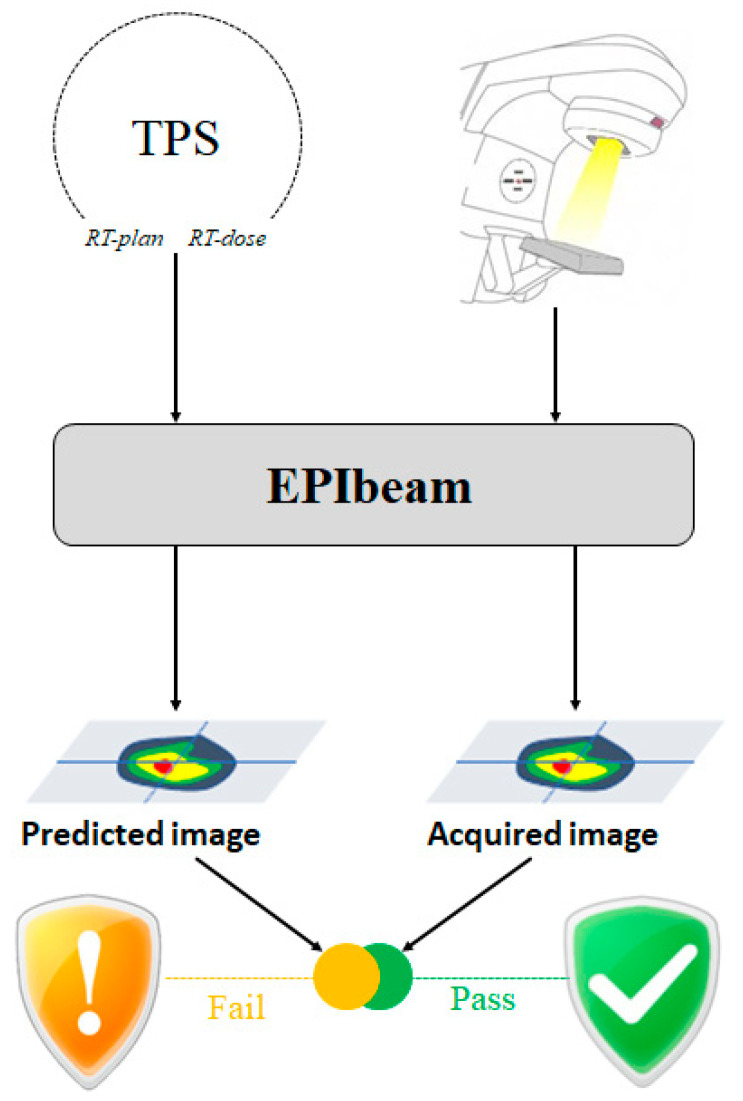
Workflow of the EPIbeam procedure.

EPIbeam (DosiSoft, Cachan, France), used by Elekta as portal dosimetry software, is EPID-based patient-specific QA software that verifies the treatment plan of treatment planning system (TPS) by comparing to the EPID image. Its workflow is shown in Figure 1. This study performed the five steps of EPIbeam (version 1.0.6.25) commissioning based on the “Beam Library: Data Preparation” procedure provided by DosiSoft [25]; the conditions of each step are shown in Table 2.

In the first step, the calibration factor was calculated through the relationship between the iso-center pixel value of EPID and the absorbed dose value calculated using Monaco TPS (version 5.51.10, Elekta).

In the second step, we corrected the ghost effect. The delay time before acquiring the next image was set differently according to MU. We set the delay time to 15 s (less than 100 MU), 120 s (100 MU), and 180 s (more than 100 MU).

In the third step, we corrected the sagging effect. Hence, we acquired the EPID image by rotating the gantry angle from 0 to 360° at 45° intervals.

In the fourth step, we acquired the EPID image for several field sizes (2 × 2, 3 × 3, 6 × 6, 10 × 10, 15 × 15, 20 × 20, and 24 × 24 cm^2^) to create a dose prediction model.

**Figure 2:**
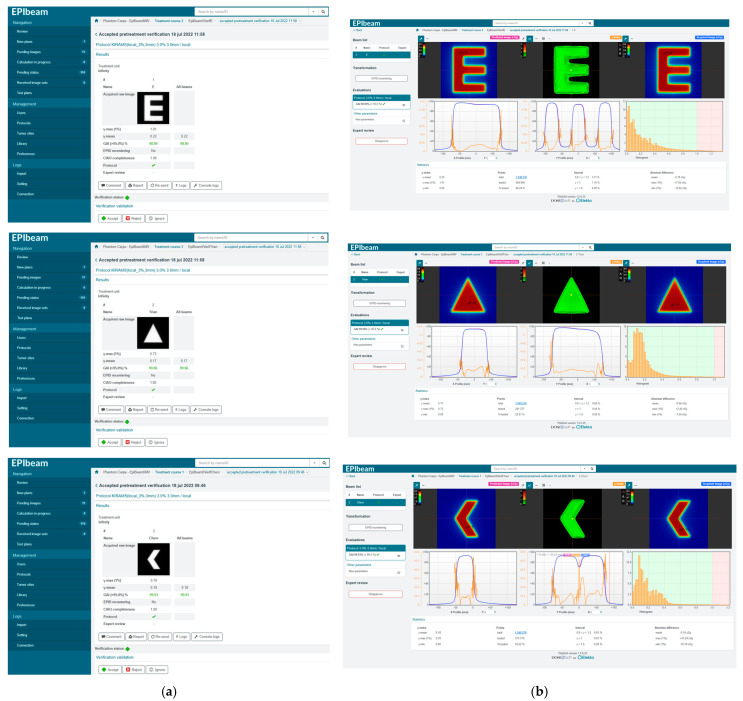
The interfaces of expert evaluation (**a**) and expert review (**b**) for each verification field recommended by DosiSoft.

The last step involved verifying EPIbeam commissioning. Figure 2 shows the interfaces about expert evaluation and review for verification field recommended by DosiSoft. For gamma analysis, the distance-to-agreement (DTA) and dose criteria were 3 mm and 3% (local), respectively, and the acceptance criterion was gamma agreement index (GAI) > 95%.

### 2.3. Clinical Implementation

Table 3 provides information on 62 cases of patient-specific QA using a 2D array detector and EPID. A PTW729 detector (PTW, Freiburg, Germany) with PTW OCTAVIUS Ⅲ phantom (PTW, Freiburg, Germany) was used as the 2D array detector in this study. In gamma analysis between TPS and measured dose maps, the PTW729 and EPID were analyzed via VeriSoft software (version 6.0, PTW, Freiburg, Germany) and EPIbeam, respectively. We applied gamma analysis criteria of 3 mm/3%, 2 mm/2%, and 1 mm/1%, and the acceptance criterion was GAI > 95%. Additionally, the gamma analysis was also performed by classifying according to the planning target volume (PTV) size to evaluate the effect of PTV size.

One package developed in the open source language R was used to perform statistical analysis between PTW729 and EPIbeam. A significant difference was considered if the *p*-value was lower than 0.05.

## 3. Results

### 3.1. Dosimetric Characteristics of the a-Si EPID

#### 3.1.1. Linearity

**Figure 3:**
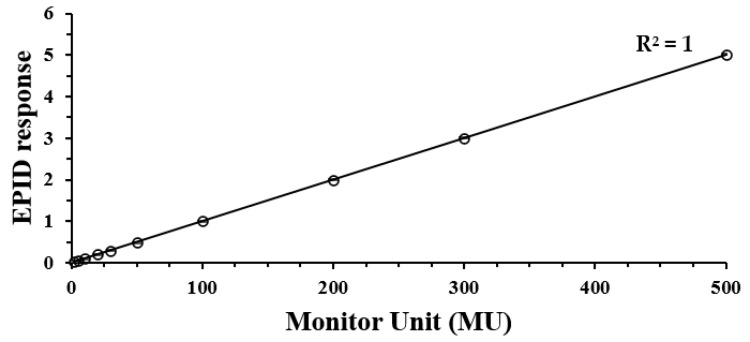
EPID linearity for 2, 5, 10, 20, 30, 50, 100, 200, 300, and 500 MU, normalized to the 100 MU response.

Figure 3 shows the EPID response according to MU normalized to 100 MU. The average difference was 0.41%; the difference of 2 MU was the highest at 1.67%, and the coefficient of determination (R^2^) value was 1.0.

#### 3.1.2. ROF Depending on Field Size

**Figure 4:**
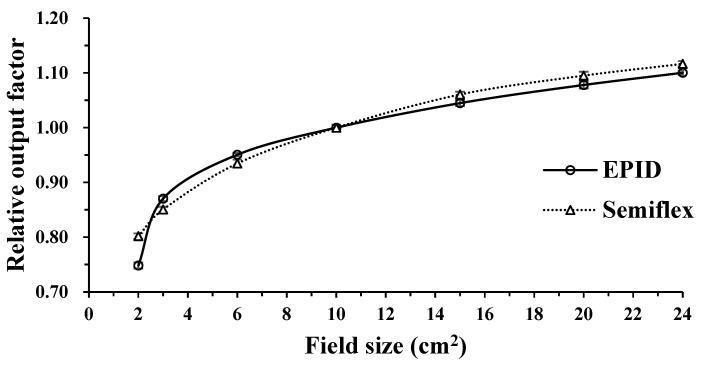
Ionization chamber and EPID relative output factor for 2 × 2, 3 × 3, 6 × 6, 10 × 10, 15 × 15, 20 × 20, and 24 × 24 cm^2^, normalized to the 10 × 10 cm^2^ output.

We measured ROF at various field sizes (2 × 2, 3 × 3, 6 × 6, 10 × 10, 15 × 15, 20 × 20, and 24 × 24 cm^2^) using a Semiflex ionization chamber and EPID. The ROF of the Semiflex ionization chamber and EPID increased with increasing field size (Figure 4) because the larger the field size, the larger the radiation scattering effect. Hence, the ROF of the Semiflex ionization chamber and EPID were highest at 24 × 24 cm^2^—the largest field size in this study—with values of 1.10 and 1.12, respectively. At 2 × 2 cm^2^, the ROF of the Semiflex ionization chamber and EPID differed the most, with ROF of 0.75 and 0.80, respectively. Additionally, the ROF showed that if the field size was smaller than 10 × 10 cm^2^, the ROF of EPID was higher, except for 2 × 2 cm^2^. By contrast, the ROF of the Semiflex ionization chamber was higher if it was larger than 10 × 10 cm^2^.

#### 3.1.3. Dose Rate Dependency

The dose rate was varied from 100 to 600 MU/min with 100 intervals; it was normalized to 400 MU/min. The maximum difference was 0.32% at 200 MU/min, and the average difference was 0.18% (Table 4).

#### 3.1.4. Beam Profile: Flatness and Symmetry

Table 5 describes the flatness and symmetry of the profile measured by PTW BeamScan and EPID. The difference in flatness was 1.06% (in-line) and 0.56% (cross-line); symmetry was −0.72% (in-line) and −0.96% (cross-line).

### 3.2. EPIbeam Commissioning Verification

We performed the gamma analysis for three verification fields (E field, triangle field, and chevron field) to test the successful commissioning of EPIbeam. All fields passed based on 3 mm/3% (local), GAI > 95%. The gamma passing rates of the E field, triangle field, and chevron field were 98.90%, 99.96%, and 99.93%, respectively (Table 6).

### 3.3. Clinical Implementation

#### 3.3.1. Gamma Analysis

**Figure 5:**
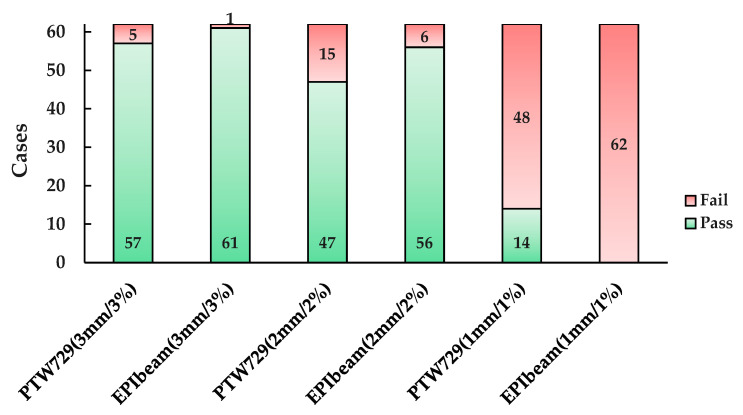
Statistics of patient specific QA based on PTW729 and EPIbeam at 3 mm/3%, 2 mm/2%, and 1 mm/1%.

First, the gamma analysis was performed by applying the three criteria (3 mm/3%, 2 mm/2%, and 1 mm/1%) for 62 cases; the number of pass or fail cases is shown in Figure 5. At 3 mm/3% (local), PTW729 had 57 cases of pass and 5 cases fail and the average gamma passing rate was 98.73 (±2.41)% (Table 7).

The five failed cases had the following commonality: the target was located outside the planning center. In particular, the gamma passing rate of Case 21, which had physically separated double targets, was the lowest (89.70%). The other four cases followed the VMAT plan. There was a difference at the edge of the dose map, which was the low-dose area.

By contrast, the EPIbeam had 61 pass cases and one fail case, with an average gamma passing rate of 99.60 (±1.31)% (see Table 7). The gamma passing rate of the failed case (Case 22) was 90.78%, with an IMRT plan with a large PTV (1499.70 cm^3^) in the L-spine.

Tighter gamma criteria resulted in the average gamma passing rates of PTW729 and EPIbeam of 96.66 (±4.46)% and 97.91 (±3.43)%, respectively, at 2 mm/2% (local). At 1 mm/1% (local), the average gamma passing rates of PTW729 and EPIbeam were 88.41 (±7.72)% and 74.87 (±9.78)%, respectively (Table 7). Notably, all cases that were analyzed with EPIbeam failed (Figure 5). The differences in the average gamma passing rates between PTW729 and EPIbeam were 0.87% (3 mm/3%), 1.25% (2 mm/2%), and 13.52% (1 mm/1%).

The cases were divided based on 150 cm^3^—the median size among 62 cases—to evaluate the effect of PTV size. As a result of applying 3 mm/3%, when the PTV was smaller than 150 cm^3^, the average gamma passing rates of PTW729 and EPIbeam were 99.55 (±1.12)% and 99.91 (±0.13)%, respectively, and they were 97.91 (±2.99)% and 99.28 (±1.79)% when the PTV was larger than 150 cm^3^ (Table 8).

#### 3.3.2. Statistical Analysis

The statistical analysis between PTW729 and EPIbeam based on gamma criteria resulted in *p*-values of 0.015 (3 mm/3%), 0.084 (2 mm/2%), and <0.01 (1 mm/1%). Furthermore, *p*-values of 0.087 (PTV < 150 cm^3^) and 0.036 (PTV > 150 cm^3^) were obtained as a result of a statistical analysis based on PTV size.

## 4. Discussion

We determined the dosimetric characteristics of the a-Si EPID prior to evaluating the suitability of the EPIbeam. The items were linearity, the ROF depending on field size, dose rate dependency, and beam profile. For linearity, the average difference with increasing MU was 0.41%, and R^2^ was 1.0. The EPID response was found to be linear based on MU. Subsequently, the ROF, depending on the field size of the EPID, was compared to that of the Semiflex ionization chamber. The ROF agreed well with the field size equal to and smaller than 4 × 4 cm^2^. However, the difference was approximately 6% at a 2 × 2 cm^2^. Mohammad et al. [12] reported a similar result. They noted that the uncertainty of the ROF in small field-size dosimetry was high because the position error was significantly affected and the number of pixels adjacent to the central axis pixel affecting the output factor measurement decreased with the field size [12]. The average difference for dose rate dependency was 0.18%. We found the response to be uniform, irrespective of dose rate. Finally, the profile measured using EPID was compared to the profile of the PTW beam scan. The difference between flatness and symmetry was approximately 1%, implying that the two profiles agreed well. Furthermore, we verified the EPIbeam commissioning using the three fields. All three fields showed high gamma passing rates of approximately 99%; hence, we found that EPIbeam commissioning was good for 6 MV.

The clinical implementation was performed to evaluate the suitability of EPIbeam for 62 cases with the IMRT or VMAT plan. The patient-specific QA results showed that, except for the 1 mm/1%, the average gamma passing rate of EPIbeam was higher than that of PTW729 and had a lower standard deviation. The QA result according to PTV size was 3 mm/3% and the averaging gamma passing rate of EPIbeam was also higher whether the PTV size was larger or smaller than 150 cm^3^.

Statistical analysis based on gamma criteria showed that the *p*-values of 3 mm/3%, and 1 mm/1%, were less than 0.05. Furthermore, the *p*-value according to PTV size was less than 0.05 when PTV was larger than 150 cm^3^. These results may demonstrate the significant difference between PTW729 and EPIbeam at 3 mm/3% and 1 mm/1%, as well as when PTV was larger than 150 cm^3^.

**Figure 6:**
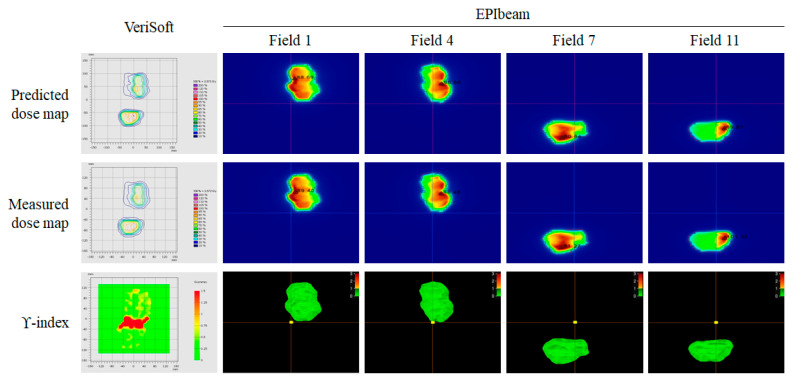
Gamma analysis of VeriSoft and EPIbeam for No. 21 case.

In Cases 21 and 22, the pros and cons of EPIbeam were clearly seen. The gamma passing rates of Case 21 had double targets of 89.70% (PTW729) and 99.79% (EPIbeam). As shown in Figure 6, the VeriSoft was included in the gap between the two targets, which negatively affected the gamma analysis. However, the gamma passing rate could be accurately calculated without the effect of the gap between targets because EPIbeam performed the gamma analysis for each field.

The limitation of EPIbeam was observed in Case 22; it had a large PTV. The gamma passing rates of PTW729 and EPIbeam were 100.0% and 90.78%, respectively. The large field size was applied to cover the large PTV. However, it was out by approximately 5 cm in the y-jaw direction from the detection area of EPID.

In summary, we successfully commissioned EPIbeam and confirmed its efficacy through clinical implementation. However, if the field size is greater than 25 × 25 owing to large PTV, the patient-specific QA should be performed using the PTW729 detector with PTW OCTAVIUS Ⅲ phantom, which has a small radiation scattering effect owing to measuring it from a close distance.

## 5. Conclusions

This study compared the PTW729 detector for IMRT and VMAT plans with various tumor sites to evaluate the suitability of EPIbeam as a patient-specific QA tool. The gamma passing rate of EPIbeam was higher than that of PTW729 at 3 mm/3%, with a statistically significant difference. Moreover, a significant difference was also observed when the PTV was greater than 150 cm^3^. Additionally, using EPID has advantages, such as a short setup time, a reduced volume average effect, and a high spatial resolution. Therefore, we consider EPIbeam to be a suitable tool for patient-specific QA along with the existing physical phantom tool.

## Figures and Tables

**Table 1:** Measurement condition for evaluation of dosimetric a-Si EPID compared to ionization chamber.

Tool	SSD or SDD	Field Size (cm^2^)	MU	Dose Rate (MU/min)
2.A.1 Linearity				
EPID	160 cm	10 × 10	2–500	400
2.A.2 Relative output factor depending on field size				
Ionization chamber	100 cm (depth 10 cm)	2 × 2 to 24 × 24	100	400
EPID	160 cm			
2.A.3 Dependency on dose rate				
EPID	160 cm	10 × 10	100	100–600
2.A.4 Beam profile: flatness and symmetry				
Ionization chamber	100 cm (depth 5 cm)	10 × 10	100	400
EPID	160 cm			

**Table 2:** Measurement condition for EPIbeam commissioning.

	Gantry Angle	Field Size (cm^2^)	MU
[Fixed condition] Energy = 6 MV, SDD = 160 cm, Dose rate = 400 MU/min			
Calibration	0°	10 × 10	100
Ghosting	0°	10 × 10	2–500
Sagging	0–315° (interval 45°)	20 × 20	100
Dose prediction	0°	2 × 2 to 24 × 24	100
Verification test	0°	10 × 10	100

**Table 3:** Characteristics of RT-plans evaluated in this study.

Tumor Site	Cases	
	IMRT	VMAT
Abdomen	1 (Case 1)	4 (Case 2–5)
Brain	-	1 (Case) 6
C-spine	2 (Case 7–8)	-
Esophagus	-	3 (Case 9–11)
Femur	2 (Case 12–13)	-
Foream	2 (Case 14–15)	-
Hypopharynx	-	2 (Case 16–17)
Humerus	1 (Case 18)	-
Liver	-	2 (Case 19–20)
L-spine	4 (Case 21–24)	-
Lung	1 (Case 25)	-
Lymph node	1 (Case 26)	3 (Case 27–29)
Mediastinum	-	3 (Case 30–32)
Neck	-	4 (Case 33–36)
Nasopharynx	-	6 (Case 37–42)
Pancreas	-	2 (Case 43–44)
Pelvic	3 (Case 45–47)	3 (Case 48–50)
Prostate	-	5 (Case 51–55)
Rectum	-	1 (Case 56)
Rib	4 (Case 57–60)	-
Scapular	1 (Case 61)	-
T-spine	1 (Case 62)	-
Total	23	39
	62	

**Table 4:** Pixel value according to dose rate for 6 MV.

Dose rate (MU/min)	100	200	300	400	500	600
Normalized pixel value	99.88	99.68	99.91	100.0	99.80	99.83
Difference (%)	0.12	0.32	0.09	0.0	0.20	0.17

**Table 5:** Flatness and symmetry of profile measured Semiflex 3D and EPID for 6 MV.

	In-Line		Cross-Line	
	Semiflex 3D	EPID	Semiflex 3D	EPID
Flatness	102.59	103.68	103.02	103.60
Symmetry	100.80	100.07	100.95	99.98

**Table 6:** Gamma analysis results of commissioning verification plans for 6 MV (3 mm/3%).

	EPIbeam vs. TPS (3 mm/3%)
E field	98.90
Triangle field	99.96
Chevron field	99.93

**Table 7:** Gamma analysis results of PTW729 and EPID patient-specific QA.

		PTW729 vs. TPS	EPIbeam vs. TPS	*p* Value
3 mm/3%	Mean	98.73	99.60	0.015
	S.D	2.41	1.31	
	Max	100.0	100.0	
	Min	89.70	90.78	
2 mm/2%	Mean	96.66	97.91	0.084
	S.D	4.46	3.43	
	Max	100.0	99.99	
	Min	82.60	79.27	
1 mm/1%	Mean	88.41	74.87	<0.01
	S.D	7.73	9.78	
	Max	100.0	89.71	
	Min	68.80	50.36	

**Table 8:** Gamma analysis results of PTW729 and EPID patient-specific QA according to PTV.

		PTW729 vs. TPS	EPIbeam vs. TPS	*p* Value
PTV < 150 cm^3^	Mean	99.55	99.91	0.087
	S.D	1.12	0.13	
	Max	100.0	100.0	
	Min	95.30	99.45	
PTV > 150 cm^3^	Mean	97.91	99.28	0.036
	S.D	2.99	1.79	
	Max	100.0	100.0	
	Min	89.70	90.78	

## Data Availability

The data presented in this study are available upon request from the corresponding author.
